# A Comprehensive Review of Hypertension in Pregnancy

**DOI:** 10.1155/2012/105918

**Published:** 2012-05-23

**Authors:** Reem Mustafa, Sana Ahmed, Anu Gupta, Rocco C. Venuto

**Affiliations:** ^1^Division of Nephrology, Department of Medicine, State University of New York at Buffalo, Buffalo, NY 14215, USA; ^2^Renal Department, Erie County Medical Center, Buffalo, NY 14215, USA

## Abstract

Hypertension is the most common medical disorder encountered during pregnancy. Hypertensive disorders are one of the major causes of pregnancy-related maternal deaths in the United States. We will present a comprehensive update of the literature pertinent to hypertension in pregnancy. The paper begins by defining and classifying hypertensive disorders in pregnancy. The normal vascular and renal physiological changes which occur during pregnancy are detailed. We will summarize the intriguing aspects of pathophysiology of preeclampsia, emphasizing on recent advances in this field. The existing diagnostic tools and the tests which have been proposed for screening preeclampsia are comprehensively described. We also highlight the short- and long-term implications of preeclampsia. Finally, we review the current management guidelines, goals of treatment and describe the potential risks and benefits associated with various antihypertensive drug classes. Preeclampsia still remains an enigma, and the present management focuses on monitoring and treatment of its manifestations. We are hopeful that this in depth critique will stimulate the blossoming research in the field and assist practitioners to identify women at risk and more effectively treat affected individuals.

## 1. Introduction

Hypertension is the most common medical disorder of pregnancy and is reported to complicate up to 1 in 10 gestations and affects an estimated 240,000 women in the United States every year [1]. Although physicians for millennia have recognized preeclampsia, relatively little is known about its pathogenesis and prevention. The primary concern about elevated blood pressure relates to the potential harmful effects on both mother and fetus. These potential adverse effects range in severity from trivial to life threatening. 

## 2. Classification of Hypertensive Disorders of Pregnancy

The National High Blood Pressure Education Program of the NHLBI classifies hypertensive disorders of pregnancy into following categories: gestational hypertension, chronic hypertension, preeclampsia, and preeclampsia superimposed on preexisting hypertension [1] (Table 1).

 Hypertension in pregnancy is defined as a systolic of 140 mm Hg or greater or a diastolic of 90 mm Hg or greater. Blood pressure should be taken in the upper arm with the patient seated using an appropriately sized cuff. The patient should be at rest for at least several minutes. The blood pressure should be confirmed with another reading at least at a twenty-minute interval or even on a separate occasion. The diastolic reading is determined by the disappearance of sound and *not* the *change* in sounds. Controversy remains as to the blood pressure criteria used to define preeclampsia. Some experts of this specialized area of medicine have argued that a rapid rise in blood pressure of 30 mm Hg systolic or 15 mm Hg diastolic should be sufficient to diagnose preeclampsia. However, the current recommendations of the 2000 working group suggest that women who experienced only this change are not yet preeclamptic but do warrant close observation, especially if this finding is accompanied by proteinuria and hyperuricemia [2].

### 2.1. Vascular Physiology of Normal Pregnancy

**Figure 1:**
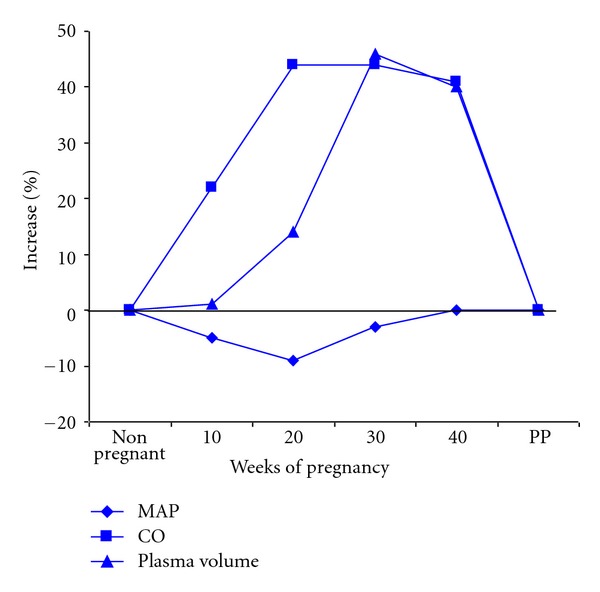
Relative changes in renal hemodynamics during normal human pregnancy. Dramatic changes occur in systemic hemodynamics during physiologic pregnancy. In uncomplicated pregnancy, mean arterial pressure drops, reaching its nadir between the 16th and 20th weeks of gestation. After the 20th week, mean arterial blood pressure slowly returns to close to pre-pregnancy levels at about 40-week gestation. Changes in systemic blood pressure are paralleled by a change in cardiac output which increases dramatically. The apex is reached between the 16th and 20th weeks of gestation. Plasma volume increases substantially as well but lags behinds the increased cardiac output. MAP: mean arterial pressure. CO: cardiac output.

Dramatic physiologic changes occur in systemic hemodynamics during pregnancy. It is essential that these differences from the nonpregnant state be appreciated when one attempts to assess blood pressure during pregnancy. In uncomplicated pregnancy, mean arterial pressure drops, reaching its nadir between the 16th and 20th weeks of gestation (Figure 1). The decline in diastolic pressure is somewhat greater than that in systolic pressure. The reduction is typically 8–10 mm Hg or just less than a 10% decline from pre-pregnancy levels. The fall in blood pressure begins with the luteal phase of menstruation and progresses if conception follows. After the 20th week, mean arterial blood pressure slowly returns to prepregnancy levels at about 40-week gestation. The circadian changes in blood pressure are maintained during pregnancy as demonstrated by ambulatory blood pressure monitoring.

Changes in systemic blood pressure are paralleled by a change in cardiac output, which increases dramatically. The apex is reached between the 16th and 20th weeks of gestation, and at its apogee the increment is typically at least 40% greater than the baseline. Both stroke volume and heart rate increase to achieve this profound rise in the quantity of blood pumped into the pulmonary and systemic circulations [3]. The volume load increase in the heart results in left ventricular hypertrophy that is commensurate with the greater amount of cardiac work required to achieve the increase in cardiac output [4]. The reduction in mean arterial pressure is even more dramatic when placed in the context of the change in cardiac output. Not only does the cardiac output increase, but also plasma volume increases substantially as well (Figure 1). This increased capacity of the circulation with a diminished tone has led to description of the vasculature as flaccid during gestation. The reduced smooth muscle tone is not limited to the vasculature, but is shared, for example, with the smooth muscle of the gastrointestinal and urinary tracts.

The circulating levels of the hormones that help regulate blood volume, specifically all components of the renin-angiotensin aldosterone system as well as catecholamines, are paradoxically increased during gestation. The usual physiologic stimuli for the release of these hormones are a reduction in plasma volume or diminished renal perfusion. Nonetheless, enhanced activity of the renin-angiotensin axis is a hallmark of the volume-expanded state of gestation. This has led to the description of pregnancy as a state of “decreased effective plasma volume.” Increases in both arterial compliance and venous capacitance appear to underlie this unique physiologic phenomenon [3], the understanding of which remains enigmatic. As will be discussed subsequently, it is a reversal of this pattern that characterizes the specific form of hypertension in pregnancy known as preeclampsia.

**Figure 2:**
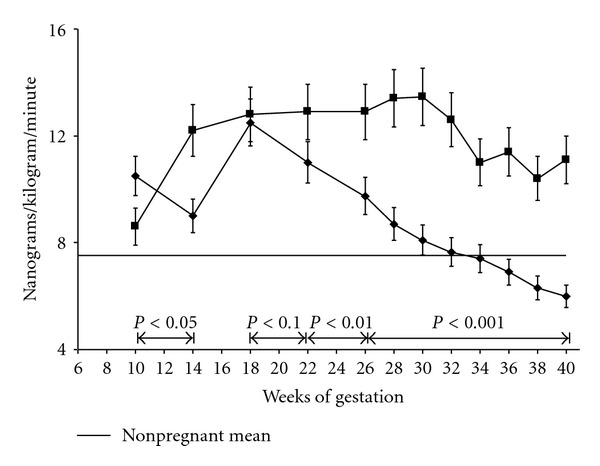
The amount of angiotensin required to raise blood pressure by 20 mm Hg. This figure demonstrates two important findings obtained from serial observations in primiparas. Women undergoing physiologic pregnancy (■) become resistant to the pressor effect of infused angiotensin II by 14 weeks of gestation. They require significantly higher dose of angiotensin II to increase blood pressure by 20 mm of Hg. In contrast, women destined to develop preeclampsia (♦) regain their sensitivity to angiotensin II between 22–26 weeks of gestation, well before any other clinical manifestations of preeclampsia are appreciated [7].

The alterations in vascular reactivity are not limited to the responses to endogenous hormones. The vasoconstrictive effect of infused pressor compounds is also substantially diminished. During gestation, pregnant women were demonstrated to be resistant to the pressor effect of angiotensin II and norepinephrine more than 40 years ago [5, 6]. Subsequently, Gant and associates demonstrated a sequential increment in the resistance to angiotensin II as pregnancy progressed, which peaked between 24–30 weeks of gestation [7] (Figure 2).

### 2.2. The Kidney in Normal Pregnancy

**Figure 3:**
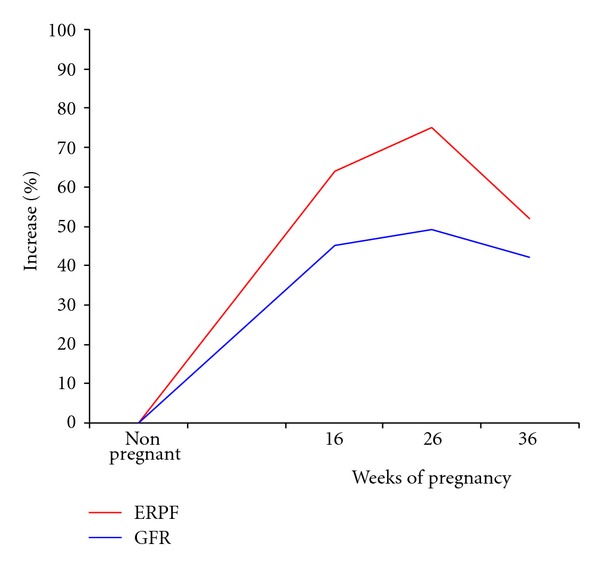
Changes in renal function during pregnancy. Kidney function also dramatically increases during pregnancy. The rapid developing rise in renal blood flow and glomerular filtration rate were documented in careful studies undertaken in humans. These increments average between 40 and 50%. Dr. Davison and his associates found that these improvements in renal hemodynamics occurred even prior to the changes in cardiac output and plasma volume. GFR: glomerular filtration rate. ERPF: effective renal plasma flow [8].

Healthy pregnant women show marked glomerular hyperfiltration. The rapid developing rise in renal blood flow and glomerular filtration rate were documented in careful studies undertaken in humans [8]. GFR begins to increase in the first trimester of pregnancy and peaks in second half of pregnancy, wherein it is increased above normal, nongravid levels by 40–60%. Davison and his associates found that these improvements in renal hemodynamics occurred even prior to the changes in cardiac output and plasma volume (Figure 3). This suggests that the mechanisms underlying these profound physiologic alterations may differ from each other or at least are not interdependent. There is no other instance in biology where such a sustained improvement of function occurs. The magnitude of the change has led many investigators to attempt to define the mechanism that underlies it so that it might be employed to treat other human conditions. Thus far no definitive explanation has been proven. If pregnancy remains uncomplicated, pregnant patients with underlying renal disease usually experience an improvement in function that is proportional to their baseline level. The reason for the physiologic change presumably is teleological and designed to accommodate the additional waste products consequent to the enlarging uterus, placenta, and fetus. Although it tends to tail off toward the end of gestation, a substantive increase in both glomerular filtration rate and renal plasma flow is sustained throughout the pregnancy. The enhanced renal function is accompanied by a reciprocal reduction in blood urea nitrogen (BUN) and serum creatinine tests commonly employed to estimate glomerular filtration rate. Low blood levels of these nitrogenous waste products are hallmarks of physiologic pregnancy (Table 2). It is critical to be cognizant of these differences from the normal nonpregnant values since subtle deviations from the pregnancy levels might presage the diagnosis of preeclampsia. 

Throughout pregnancy the average women will retain about 1000 mEq of sodium as she experiences the fairly steady increase in extracellular and plasma volume. Nonetheless women experiencing physiologic pregnancy will respond appropriately to sodium restriction or sodium infusion [3].

### 2.3. Volume and Hemodynamic Alterations in Preeclampsia

It is difficult to study totally untreated preeclampsia, and often preeclampsia is diagnosed in patients with underlying chronic medical conditions. Data generated from treated preeclamptic patients or those patients with preexisting renal disease, diabetes, or hypertension might not accurately reflect those of the uncomplicated preeclamptic patient. These concerns aside, the available data suggests that the systemic hemodynamic preeclamptics vary substantially from those of women with uncomplicated pregnancy.

Visser and Wallenburg undertook detailed hemodynamic assessments of untreated primiparous preeclamptics. Using Swan-Ganz catheters, they consistently found cardiac outputs and intravascular volumes lower and systemic vascular resistance and cardiac afterload higher in these carefully selected group of women with pregnancy-induced hypertension as compared to normal control pregnant subjects [3].

If one focuses on the properties of the arterial system in preeclampsia using impedance techniques, compliance of the large conduit arteries is reduced. This suggests that the reservoir properties of the arterial system are compromised. The left ventricle muscle mass and cardiac wall diastolic pressure in late gestation is similar between preeclamptic and controlled subjects. Limited data suggests, however, that left ventricular contractility in preeclamptics is inappropriately low given the high afterload [3].

Some changes in the systemic hemodynamics of pregnant women destined to become preeclamptic may develop prior to overt clinical manifestations of the disease. Ambulatory blood pressure readings suggest that a reduction or obliteration in the usual decrease in nocturnal blood pressure may be present in many patients who eventually become preeclamptic. Such changes usually manifest at 18 to 26 weeks of gestation. The resistance to pressor substances appears to be altered well before the systemic hypertension and proteinuria are noted. Figure 2 shows that the sensitivity to the pressor effect of infused angiotensin changes in women destined to become preeclamptic. These individuals exhibit sensitivity similar to that seen in nonpregnant women well before they clinically manifest preeclampsia. In contrast, claims that high cardiac output necessarily precedes the development of preeclampsia appear to be based on an insufficient database [3].

Renin levels actually decrease in preeclamptic patients, but remain well above those of nonpregnant individuals. Similar changes are also seen in the circulating levels of aldosterone and angiotensin II. Maintaining relatively high levels of these hormones may be critically important because most often preeclamptic patients have a relatively diminished plasma volume.

### 2.4. Renal Alterations in Preeclampsia

The dramatic improvement in renal function experienced by women undergoing a physiologic pregnancy is abrogated in women who develop preeclampsia. The GFR and renal blood flow decline. The severity of the reduction is quite variable and correlates with the overall severity of the illness. If proteinuria develops, as is most common, and a kidney biopsy were to be undertaken, it would typically show glomerular endotheliosis. This lesion, although not limited to pregnancy, is characteristic in preeclamptic women. This endothelial abnormality is quite consistent with the notion of endothelial injury playing a key role in the pathophysiology of this systemic condition with the kidney not being spared. These hemodynamic and endothelial changes also appear to make the kidneys more vulnerable for the development of acute renal failure (acute tubular necrosis) and uncommonly a particular form of acute, often irreversible renal failure known as renal cortical necrosis. Cortical necrosis is seen almost exclusively in severe preeclamptics and rarely occurs in settings outside of pregnancy [9].

### 2.5. Pathophysiology of Preeclampsia

The pathophysiology of de novo hypertension and proteinuria in pregnancy known as preeclampsia remains largely undiscovered. More than 30 years ago Dr. Leon Chesley, a champion in the field of hypertension in pregnancy divided the most likely causative factor into four major categories: dietary, renal, immunologic, and placental [10]. Subsequently, the evidence that poor diet or preexisting renal abnormalities underlie the majority of episodes of preeclampsia has not been sustained. The role of immunologically mediated vascular injury, as the initiating cause remains plausible and will be explored.

Delivery of the placenta usually initiates the resolution of the acute clinical symptoms of preeclampsia, suggesting that the placenta plays a central role in preeclampsia pathogenesis. During normal pregnancy, the placenta undergoes dramatic vascularization to enable circulation between fetus and mother. Placental vascularization involves vasculogenesis, angiogenesis, and pseudovasculogenesis or maternal spiral artery remodeling. These processes require a delicate balance of proangiogenic and antiangiogenic factors. The imbalance of proangiogenic and antiangiogenic factors in preeclampsia is thought to trigger abnormal placental vascularization and disease onset.

Underlying genetic explanations for the overproduction of anti-angiogenic factors in preeclampsia are still being proposed [11].

### 2.6. The Role of Uteroplacental Ischemia

Altered uteroplacental blood flow has long been the focus of the pathophysiology of preeclampsia. Clinicians and research scientists have garnered a wealth of data to support the hypothesis that a reduction in uterine blood flow is the overriding factor in the etiopathogenesis of this condition (Table 3). The placentae of women with preeclampsia are uniformly abnormal. The primary pathology appears to be at the maternal-fetal interface and is characterized by poor trophoblastic invasion of the uterus. The endovascular invasion of the spiral arteries is incomplete. Specifically, the failure of the cytotrophoblasts to penetrate deep and cause a “widening of the pathway” appears to explain the relative reduction in uteroplacental blood flow. Additional pathologic findings include placental infarcts. It is not surprising; consequently, that intrauterine growth retardation is frequently associated with preeclampsia. Virtually all clinical settings that favor the development of preeclampsia also favor the possibility that growth of the intrauterine contents outstrips the capacity to commensurately improve blood supply. 

Finally, there is a wealth of data derived from experiments in various pregnant mammals spanning the spectrum from rats to primates supporting this hypothesis. When subjected to reduced uteroplacental blood flow, these animals develop findings that mimic those seen in preeclamptic women. Sustained hypertension, proteinuria, and glomerular endotheliosis, the renal lesion that characterizes preeclampsia, have all been reported in these laboratory animals following uterine constriction of blood flow [12]. 

### 2.7. Maternal Endothelial Dysfunction

Although preeclampsia appears to originate in the placenta, the tissue affected most is the maternal endothelium. The clinical manifestations of preeclampsia reflect widespread endothelial dysfunction, with vasoconstriction and end organ ischemia. Systemic hypertension, renal, hepatic, and cerebral vascular pathology are hallmarks of severe preeclampsia. Taylor, Davidge and Roberts explore in great depth the evidence placing endothelial dysfunction as the focal point of the disease [13]. They point out that endothelial “activation” and dysfunction are reflected in the inappropriate vasoconstriction and its propensity toward a hypercoagulable state and the widespread microvascular thrombi, most notably that are seen nearly uniformly in the placenta of preeclamptics. These investigators suggest that endothelial dysfunction may be manifested by the altered synthesis and release of endothelial cell products. Among the various compounds, which act on the endothelium, are the prostanoids and nitric oxide. Nitric oxide synthesis is increased in women undergoing physiologic pregnancy, whereas analysis of tissue and urine samples strongly suggest that nitric oxide production is impaired in preeclamptic women. In laboratory animals nitric oxide synthase inhibition can produce a condition, which bears many similarities to preeclampsia [12]. Likewise, there appears to be a role for possible imbalance between vasodilating and vasoconstricting prostaglandins. Synthesis of the vasorelaxant prostacyclin increases in physiologic pregnancies, whereas more of the vasoconstrictor thromboxane is produced in women whose pregnancies are complicated by high blood pressure and proteinuria. Whether these particular compounds play a primary role or are only a part of the progression of the pathophysiology is unclear. A treatment strategy, nonetheless, was devised using low-dose aspirin to attempt to confirm the relationship between thromboxane and vasodilating prostaglandins, since low-dose aspirin may selectively inhibit thromboxane synthesis. The results of studies on a large number of primiparous women who were not at high risk to develop preeclampsia failed to support a benefit from this strategy. Some advocates, however, still hold to the notion that selective treatment of women who are at extremely high risk because of preexisting hypertension or renal disease for example, may be of benefit. 

### 2.8. Antiangiogenic Factors in Preeclampsia

There is a published body of work that has grown almost logarithmically since 2003 suggesting that circulating angiogenic factors play a key role in the pathogenesis of preeclampsia. Increased expression of soluble fms-like tyrosine kinase (sFlt1), together with decreased placental growth factor (PGF) and vascular endothelial growth factor (VEGF) signaling, were the first abnormalities described [14].

#### 2.8.1. sFlt1: A Circulating Antagonist to VEGF and PGF

Several investigators spearheaded by Karumanchi have seized on the finding that pregnant women may produce a soluble variant of vascular endothelial growth factor receptor. This kinase has been designated sFlt1. sFlt1 consists of the extracellular ligand-binding domain of Flt1, but lacks the transmembrane and intracellular signaling domain. Hence, it is secreted into the circulation where it binds and antagonizes both vascular endothelial growth factor (VEGF) and placenta growth factor (PGF) [15]. Both are potent stimuli for the vascular expansion essential to the development of the uteroplacental unit and act via their effects on endothelial cells [16]. Even more recent clinical evidence has been gathered that supports the notion that both circulating and placental levels of this soluble receptor blocker are higher in women with preeclampsia than in women with uncomplicated pregnancy. These hypertensive women also have been demonstrated to have lower levels of PGF and VEGF. Circulating levels of sFlt1 and PGF are altered several weeks before the onset of clinical disease and are correlated with severity of the disease [17, 18]. sFlt1 levels normalize several days after delivery, coinciding with improvement in proteinuria and hypertension. Support for the key role of this kinase in the pathophysiology of preeclampsia has been garnered from studies undertaken in a baboon model of hypertension in pregnancy induced by uteroplacental ischemia [19]. In these primates with restricted uterine arteries, a temporal link was observed between the onset of hypertension and renal dysfunction and the increase level of the kinase. This rise in the kinase was also correlated with the blunted effectiveness of PGF and VEGF. Based on the findings in preeclamptic women, assays that measure s-Flt, PGF, and VEGF have been touted as potential tools to differentiate preeclampsia from other categories of hypertension in pregnancy.

More recent studies have identified a second sFlt1 splice form expressed in cytotrophoblasts, which differs in its c-terminus and also appears to be upregulated in preeclampsia [20]. The biologic significance of the different sFlt1 variants with regards to anti-angiogenic activity and their role in the pathogenesis of preeclampsia is a subject of ongoing study. Selectively removing soluble FMS-like tyrosine kinase, using an extracorporeal adsorption technique, reduced proteinuria, stabilized blood pressure, and permitted prolongation of pregnancy in a small group of women with preeclampsia very early in pregnancy. This observation supports the notion that this protein kinase has a role in the pathophysiology of preeclampsia [21].

#### 2.8.2. Soluble Endoglin: A Circulating Antagonist to Transforming Growth Factor-B

Proponents of the vascular endothelial growth factor-receptor antagonist hypothesis or so-called anti-angiogenic theory recognize that blocking the action of these growth factors alone was insufficient to explain all the clinical manifestations seen in severe eclampsia. Another factor, soluble endoglin (sEng), has now been also found to be upregulated in preeclampsia in a pattern similar to sFlt1. sEng is a truncated form of endoglin (CD 105), a cell surface receptor for transforming growth factor B (TGF-B), which binds and antagonizes TGF-B [22]. This compound not only potentiates the anti-angiogenic actions of s-Flt-1 kinase, but ultimately results in the decreased production of nitric oxide. This type of endothelial abnormality would be requisite to account for the disseminated intravascular coagulation and the other hematologic components seen in patients with severe preeclampsia.

As with sFlt1, circulating sEng levels are increased weeks before preeclampsia onset, and increased sEng levels are observed in the reduced uterine perfusion pressure rat model of preeclampsia [23]. Cultured placental trophoblasts from women with preeclampsia show increased sEng and sFlt1 expression, both at normoxic conditions and in response to hypoxia, as compared with normal placental trophoblasts [24].

 Endoglin excess has now been incorporated into the anti-angiogenic theory. Collectively, this is an appealing hypothesis. Skeptics could say, however, we may not as yet have reached the root cause level.

## 3. Relaxin in Pregnancy

Relaxin, a peptide hormone secreted by the corpus luteum, circulates during pregnancy in human beings, nonhuman primates, rats, and mice [25]. The hormone also is detectable in the circulation during the luteal phase of the menstrual cycle in both women and nonhuman primates [25]. Traditionally, relaxin has been investigated in the context of the reproductive tract; however, it was suggested by Hisaw et al. that relaxin has a vasodilatory role [26, 27], this was further shown in subsequent studies by St-Louis and Massicotte [28]. Relaxin administration to nonpregnant rats was shown to mimic the vasodilatory phenomenon of pregnancy [29]. Furthermore, immunoneutralization of relaxin or its elimination from the circulation during midterm pregnancy in rats prevents maternal systemic and renal vasodilation, and the increase in global arterial compliance [30]. Evidence suggests that the vasodilatory responses of relaxin are mediated by its major receptor, the relaxin/insulin-like family peptide 1 receptor, RFXP 1 [31], that is largely expressed in vascular smooth muscle [32]. The possibility that angiogenic growth factors may be secreted by the vascular smooth muscle upon RFXP1 activation is being entertained [32]. Jeyabalan et al. reported an association of low first trimester relaxin concentrations with increased risk of developing preeclampsia [33]. This study raised the possibility that these women may experience defective decidualization and trophoblast invasion or fail to adequately vasodilate in early pregnancy owing to low levels of circulating relaxin, thereby predisposing them to develop preeclampsia.

## 4. Renin Angiotensin Signaling in Preeclampsia

There is an increase in almost all the components of renin-angiotensin system during an uncomplicated pregnancy, but renin activity, angiotensin II, and aldosterone decrease in preeclampsia for reasons that are unclear. Numerous studies report the presence of angiotensin II type 1 receptor agonistic antibody (AT1-AA) found circulating in preeclamptic women [34, 35]. Many recent studies have shown that by activating AT1 receptors on a variety of cell types, these autoantibodies could increase certain factors (including sFlt1, sEng, Plasminogen activator inhibitor-1, reactive oxygen species, tissue factor, and NADPH oxidase) that lead to preeclamptic pathophysiology such as endothelial cell dysfunction and vascular damage [36, 37]. Granger et al. isolated AT-AA1 from the rats manipulated by reduction in uterine perfusion pressure. These rats also demonstrated development of hypertension, proteinuria, increased sFlt1, endothelin production, and endothelial dysfunction [38].

## 5. The Role of Alterations of the Immune System

Over the last 30 years, significant progress has been made in understanding the role of immune mechanisms in the development of preeclampsia. It remains unexplained why primiparous women are more susceptible to this condition and also why the high preeclampsia attack rate (5–7%) noted in primiparous is unchanged in women who are having a first pregnancy with a second partner. This has fostered the suspicion that the immunologic difference between the partners, embedded in the fetus triggers an immune response in pregnant women. Redman et al. have postulated that preeclampsia is the continuum of the immune-mediated inflammatory changes seen in normal pregnancy. Most investigators believe that endothelial injury, perhaps caused by cytokine release induced by inflammation is a basic mechanism underlying the pathogenesis of preeclampsia [39].

It has also been postulated that immune accommodation to the fetus needs to be learned, and this adaptation may be relatively defective in the first pregnancy leading to the higher preeclampsia attack rate which declines in but less so in subsequent pregnancies. Such conditioning might be acquired from previous pregnancy, abortion, and exposure to paternal semen and seminal plasma. Maternal exposure to fetoplacental tissues varies with gestational age, and two interfaces have been described. Interface 1, which is predominant in the first half of pregnancy, exists between maternal immune cells and invasive, extravillous HLA expressing trophoblasts in decidua. Interface 2, which predominates during the second half of pregnancy, comprises syncytiotrophoblasts that are in contact with maternal blood-borne immune cells. Syncytiotrophoblasts are HLA negative, and thus the paternal alloantigens are only expressed at interface 1, which is most active in first half of pregnancy [39].

It is tempting to suggest that those women who respond vigorously to these foreign antigens are more susceptible to develop endothelial injury that precedes preeclampsia. Women indeed often develop persistent antibodies to the fetal HLA antigens of paternal origin. The presence of these antibodies substantially increases the rate of graft rejection post transplant. It is of note that the endothelium is the major attack site of antibody-mediated rejection that develops not uncommonly in this setting.

## 6. Role of Genetics in Preeclampsia

Although the risk factors for preeclampsia are both genetic and environmental, the presence of preeclampsia in first degree relatives increases a woman's risk of preeclampsia by 2 to 4 fold [40, 41]. Genetic factors may play an important role in the angiogenic imbalance found in patients with preeclampsia. Recently, several polymorphisms in sFlt1 and VEGF have been associated with severity of preeclampsia [42]. Although circulating PGF, sFlt1, and sEng levels have been shown to be important markers of preeclampsia, no causal mutations in these genes associated with preeclampsia have been identified so far [43]. However, women with trisomy 13 fetuses have a higher incidence of preeclampsia [44], suggesting that gene dosage or copy number variation may contribute to the development of preeclampsia. Notably, the Flt1 gene is located on chromosome 13.

There is some evidence to suggest that in addition to maternal genotype, paternal (or fetal) genotype may also contribute to risk of preeclampsia. The risk of fathering a preeclamptic pregnancy is increased among males who fathered a preeclamptic pregnancy with a different partner [45]. Also, men who are born from a pregnancy complicated by preeclampsia are at a higher risk of fathering a preeclamptic pregnancy [46].

## 7. Diagnosis of Preeclampsia

The diagnosis of preeclampsia is largely based upon meeting the characteristic clinical features outlined above which define preeclampsia. In this section, we explore various tools proposed to predict the development and/or accurately diagnose preeclampsia.

### 7.1. Clinical Assessment

The hallmark features in preeclampsia include developing systolic blood pressure (SBP) ≥140, or diastolic blood pressure (DBP) ≥90, and proteinuria of 0.3 grams or greater in a 24-hour urine specimen after 20 weeks of gestation in a woman who was previously normotensive. Hypertension is generally the earliest physical abnormality seen in preeclampsia and is the most important clinical clue to the presence of the disease. Since SBP and DBP readings are an essential part of the diagnosis of preeclampsia, ensuring that the optimal and appropriate ways are employed to measure BP cannot be overemphasized.

Using different indices of BP to predict preeclampsia has been comprehensively evaluated in a meta-analysis published by Cnossen et al. [47]. This meta-analysis included 34 studies and evaluated using SBP, DBP, mean arterial pressure, and the increase over time in BP. The data from this meta-analysis supports the conclusion that BP measurements in the first and second trimesters have only a modest ability to predict preeclampsia [47].

### 7.2. Laboratory Tests

**(1) Proteinuria**

Although proteinuria is generally considered an essential characteristic of preeclampsia, preeclampsia should be suspected in any pregnant woman with hypertension and characteristic signs or symptoms, even if proteinuria is absent. Twenty percent of women who develop eclampsia have no proteinuria and 10 percent of women with other clinical and/or histological manifestations of preeclampsia have no proteinuria [48]. Women with proteinuria detected by urine dipstick should undergo quantitative measurement of protein excretion. Use of the urine protein: creatinine (P : C) ratio to estimate 24 h protein excretion for the diagnosis of preeclampsia has been controversial. The P : C ratio has been compared with 24 h urine collection in pregnant women with discordant conclusions. Though the earlier studies reflected that urine protein: creatinine ratios did not correlate with 24-hour urine protein excretion during gestation [48], the more recent literature suggests a significant correlation between these tests [49]. A meta-analysis involving 974 pregnant women from 10 studies showed a pooled sensitivity of 90% and specificity of 78% using P : C ratio cutoffs between 0.19 and 0.25, as compared with “gold standard” of 24 h urine protein excretion (>300 mg/day) [50]. Most misclassifications tended to occur in women with borderline proteinuria (250 to 400 mg/day) [51]. Hence it is reasonable to use the urine P : C ratio for the diagnosis of preeclampsia, with 24 h collection undertaken when the results are equivocal. Microalbuminuria and albuminuria, however, have a poor value to predict the subsequent development of preeclampsia [9].

**(2) Kidney Function**

The kidney is the organ most likely to manifest endothelial injury related to preeclampsia. Although the plasma creatinine concentration is generally normal or only slightly elevated (1.0 to 1.5 mg/dL (88 to 133 mmol/L)), this could represent a decrease by 30–40% of glomerular filtration rate (GFR) for the values experienced in pregnant normotensive controls (Table 1). Renal failure is an unusual complication that most often occurs in patients who develop severe preeclampsia. Distinguishing preeclampsia from an exacerbation of underlying renal disease can be challenging. This is especially true in patients with preexisting proteinuria because protein excretion almost always increases as pregnancy progresses. Preexisting renal disease is a well-described risk factor for preeclampsia, and the onset of preeclampsia in early pregnancy (before 32 weeks) is most often seen in patients with underlying kidney disease or hypertension.

**(3) Serum Uric Acid**

Hyperuricemia was purportedly among the earliest manifestation described in preeclampsia. Different theories were explored trying to explain this finding [52]. In two systematic reviews published in 2006, serum uric acid was found to be a poor predictor of preeclampsia and its complications [53, 54]. A meta-analysis by Koopmans et al. found uric acid to be useful to predict maternal complications and assist with management of pre-eclampsia [55]. First trimester elevated uric acid was associated with later preeclampsia and more strongly with preeclampsia and gestational hypertension with hyperuricemia in a prospective cohort study [56].

**(4) Urinary Calcium Excretion**

Hypocalciuria has been reported in association with preeclampsia [57]. The mechanisms for this change is not clear, but multiple studies designed to evaluate urinary calcium excretion have shown that this parameter has no predictive value in the diagnosis of preeclampsia [9].

### 7.3. Provocative Tests

Roll over test; isometric exercise test, and angiotensin II sensitivity test [58–60] were devised to demonstrate the presence of abnormally increased vascular activity during gestational weeks 28–32 and before the clinical onset of preeclampsia. None of these tests are currently being used clinically because they are expensive, time-consuming, invasive, subjective, and, most important, unreliable.

### 7.4. Doppler Ultrasonography of the Uterine Arteries

The inadequate placental perfusion has lead to the use of Doppler ultrasonography to assess the velocity of the blood flow in the uterine arteries. A persistence of an early diastolic notch after 24 weeks of gestation or abnormal flow velocity ratio's has been associated with an inadequate trophoblast invasion. Pregnancies associated with an abnormal uterine Doppler after 24 weeks of gestation (high pulsatility index and/or presence of an early diastolic notch) are associated with a more than six-fold increase in the rate of preeclampsia [61]. Among high-risk patients with a previous preeclampsia, Doppler ultrasound of the uterine arteries has an excellent negative predictive value [62].

Current data do not support the use of Doppler ultrasonography for routine screening of patients for preeclampsia [63]. However, several studies have shown that the measurement of uterine perfusion in the second trimester and analysis of angiogenic markers have a high detection rate especially for early onset preeclampsia [64, 65].

### 7.5. Biomarkers in Prediction and Diagnosis of Preeclampsia

Several markers heretofore described, might help, alone or in combination to predict and/or diagnose preeclampsia. The data, however, are derived largely from small case studies in selected population. When evaluating new screening strategies, not only sensitivity, specificity, and predictive values need to be taken into account, but costs, patient's acceptability, and quality control also must be considered.

Studies have consistently reported elevated serum levels of sFlt-1 in women with preeclampsia compared with normal pregnancies [66–68]. Levine et al. reported a mean sFlt-1 value of 4382 pg/mL in women with preeclampsia compared with 1643 pg/mL in the control group [66]. Similar values have been reported in other studies [69–71], with most concluding that the higher the sFlt-1 level, the more predictive it is of preeclampsia. Importantly, this increase in serum sFlt-1 levels may be detected up to 5 wks before the clinical onset of clinical symptoms.

Levine et al. found a mean serum PGF concentration in women with preeclampsia of 137 pg/mL compared with 669 pg/mL in controls [66]. Unfortunately, there was a substantial overlap in the sFlt-1 and PGF concentrations between patients destined to develop preeclampsia and those who will go on to have normal pregnancies. Widmer et al. also reported considerable difference in the methods of various studies and concentrations of sFlt-1 in a systematic review published in 2007 [72].

More recently, the assessment of the sFlt-1: PGF ratio in the maternal serum has been proposed as more reliable marker of overall preeclampsia risk. Preliminary data suggests that this ratio may be more accurate than sFlt-1 or PGF alone [70]. This test was launched in Europe by Roche as a second trimester screening test for preeclampsia. A financial analysis found that the cost of this test when added to the standard protocol was negated by timely management of patients who would not have been diagnosed if only existing tests had been used (false negative cases) [73].

Increased maternal serum levels of sEng were detected prior to preeclampsia onset in healthy, nulliparous women [74, 75] as well as in high-risk pregnant population [76].

A Korean study demonstrated that the combined ratio of (sFlt-1 + soluble endoglin) : (PGF + TGF beta −1) during the second trimester had the highest odds ratio and lowest false positive rate as compared to the individual markers for prediction of preeclampsia [77].

The results of various studies, unfortunately, have been inconsistent, and larger studies in more heterogenous population are needed to better define the clinical utility of these tests. There is some data, however, to suggest that tests for these biomarkers may be of use when applied to selected high-risk patients such as those with underlying hypertension [78].

The other suggested markers for the prediction or detection of preeclampsia are

- placental protein 13 (PP-13);
- pregnancy-associated plasma protein A (PAPP A);
- inhibin A;
- P-selectin;
- activin A;
- pentraxin;
- cell-free fetal DNA;
- ADAM-12;
- beta-HCG;
- 2-Methoxyestradiol (2-ME).

## 8. Consequences of Hypertension in Pregnancy

Hypertension in pregnancy is a major cause of maternal morbidity and mortality in the United States. There is approximately one maternal death due to preeclampsia-eclampsia per 100,000 live births, with a case-fatality rate of 6.4 deaths per 10,000 cases [79, 80]. The outcome of hypertension in pregnancy is, not surprisingly, affected by multiple factors. These embrace (but are not limited to) gestational age at onset, severity of disease, and the presence of comorbid conditions including diabetes mellitus, renal disease, thrombophilia, or preexisting hypertension [81]. Adverse outcomes related to hypertension in pregnancy can be divided into short-term versus long-term complications. While short-term complications can be further subgrouped into maternal and fetal complications, long-term outcomes are mainly maternal.

### 8.1. Short-Term Complications

#### 8.1.1. Maternal

Outcomes for pregnancy complicated by hypertension range from uneventful pregnancy in women with chronic, controlled hypertension to death in cases of preeclampsia-eclampsia. The major adverse outcomes include central nervous system (CNS) injuries such as seizures (eclampsia), hemorrhagic and ischemic strokes, hepatic damage ranging from transaminase elevation, the so-called “HELLP syndrome” (hemolysis, elevated liver enzymes, and low platelets), hepatic failure, renal dysfunction (spanning the gamut from a trivial reduction in glomerular filtration rate and minimal proteinuria to reversible acute renal failure or so-called acute tubular necrosis to even irreversible renal failure secondary to renal cortical necrosis) as well as increased frequency of cesarean delivery, preterm delivery, and abruptio placentae [2, 80, 81].

#### 8.1.2. Fetal

The effects of chronic, controlled hypertension in pregnancy on the fetus are minimal. However, preeclampsia-eclampsia can lead to higher frequency of induced labor, fetal growth restriction, neonatal respiratory difficulties, and increased frequency admission to neonatal intensive care unit. Hypertension in pregnancy, even in its more severe forms, causes only minimal increased risk for perinatal or fetal death [2, 82].

### 8.2. Long-Term Complications

Though hypertension in pregnancy/preeclampsia is usually thought of as a short-term problem that resolves itself with delivery, it still carries significant risk for remote complications. Those infants born small and premature may experience prolonged stays in neonatal intensive care units and often face developmental delays. Remote outcomes include the risk of preeclampsia in subsequent pregnancies and several long-term maternal health risks as described below.

#### 8.2.1. Risk of Recurrence

The risk of recurrent preeclampsia in subsequent pregnancies varies with the severity and time of onset of the acute episode [63]. It is estimated that women with severe, early preeclampsia during their first pregnancy will have a high risk of recurrent preeclampsia in their subsequent pregnancies (25–65%) [83, 84]. On the other hand, for milder forms of preeclampsia the risk of recurrent episode is still elevated, though to a lesser degree (5–7%) in comparison to women who remained normal during their first pregnancy (1%) [85–87]. The recurrence risk of preeclampsia is lower when the first pregnancy was a twin as compared to a singleton pregnancy [88].

#### 8.2.2. Cardiovascular Complications

The association between preeclampsia and cardiovascular diseases is both well described and well documented. Women with history of preeclampsia are at significantly increased risk to develop hypertension, ischemic heart disease, stroke, type II diabetes, and venous thromboembolism in comparison with women without history of the disease [89]. Factors linked to increased risk of long-term cardiovascular diseases are early onset preeclampsia, recurrent preeclampsia, severe preeclampsia, gestational hypertension, or preeclampsia with onset as a multipara [89]. Peripartum cardiomyopathy more often develops in women with preeclampsia. The pathophysiologic relation between preeclampsia and subsequent late-developing cardiovascular disease is unclear. Many hypotheses have been explored including impaired endothelial function, increased insulin resistance, sympathetic overactivity, proinflammatory activity, and the abnormal lipid profile, which usually constitute an early manifestation of metabolic syndrome [90–94].

#### 8.2.3. Renal Disease

More renal biopsies are undertaken in victims of preeclampsia than in unaffected women [95]. There is also an increased risk for women with history of preeclampsia to develop end-stage renal disease (ESRD), though the absolute risk appears to be low. A recently published study that evaluated data from the Norwegian national birth and ESRD registries found that the risk of subsequent ESRD increases with increased recurrent episodes of preeclampsia in two or more pregnancies [95].

#### 8.2.4. Cancer

Multiple observational studies evaluated the possible association between hypertension in pregnancy and cancer risk. Overall, women with preeclampsia were found to be at reduced risk or had no excess risk of cancer when followed by extended periods postpartum [96–99]. This was confirmed by a recent systematic review that found no significant association between preeclampsia and risk of cancer. This “protective” effect of preeclampsia may be explained, at least in part, by the possible role of the immune system in the disease pathogenesis. Women with responsive immune systems may be more vulnerable to develop preeclampsia but enjoy some protection from malignancy.

## 9. Treatment of Hypertension

The first principle of treatment of hypertension in pregnancy is to correctly diagnose the category (Table 1) and severity of the hypertension. Implicit to this guide is the aforementioned limited value of attempting to completely normalize the blood pressure in this setting. The second and perhaps even more important principle is to understand the potential vulnerability of the fetus to treatment.

### 9.1. Chronic Hypertension

The estimated prevalence of chronic hypertension in pregnancy in United states is 3% and has been increasing over time. This increase in prevalence has been attributed to the increased prevalence of obesity and delay in childbearing to ages, when chronic hypertension is more common [100]. Although these patients are at higher risk of superimposed preeclampsia, many will naturally experience a physiological lowering of blood pressure during pregnancy, and a reduction in the requirement for any previously prescribed antihypertensive medication. A return to blood pressure in hypertensive range in the third trimester is not unexpected. The goal of treatment is to maintain blood pressure at a level that prevents maternal cerebrovascular and cardiovascular complications. Prevention of preeclampsia is desirable; however, current evidence has not shown that either specific blood pressure targets in pregnancy, or specific antihypertensive agents modify the risk of superimposed preeclampsia in women with preexisting hypertension [101].

Women with the following conditions are at increased risk for maternal and fetal complications and should have a lower threshold for treatment [102]:

- underlying renal disease;
- secondary hypertension;
- end-organ damage (e.g., ventricular dysfunction, retinopathy);
- maternal age over forty years old;
- microvascular disease;
- history of stroke;
- previous perinatal loss;
- diabetes.

### 9.2. Gestational Hypertension

Gestational hypertension is elevated blood pressure, which develops after 20 weeks of gestation in a previously normotensive woman, though without proteinuria. It complicates 6% of all pregnancies. These women are at high risk for developing preeclampsia that can occur at any time including the first postpartum week and need close monitoring. Approximately 15–45% will eventually develop preeclampsia [103, 104]. The goal of treatment is same as chronic hypertension.

### 9.3. Preeclampsia

The general principles as outlined to guide the treatment of women with chronic hypertension are applicable to the preeclamptic patients. Close monitoring to recognize fetal distress while receiving treatment is essential. Early onset preeclampsia (less than thirty-four weeks) requires careful use of antihypertensive medications, bed rest, and in-hospital monitoring of both mother and fetus. This approach may help delay delivery and thus improve fetal outcome. Often these patients are intravascularly depleted and are more susceptible to precipitous, drug-induced drops in blood pressure. If signs of other fetal or maternal distress are noted, delivery is the definitive treatment. Concerns about hypotension and decreased uteroplacental blood flow are central to the treatment of the preeclamptic patient, since placental ischemia is the focal point of preeclampsia pathophysiology. Furthermore, lowering of BP does not reverse the primary process. The ultimate goal of antihypertensive therapy is to reduce the main risks to the mother, which include placental abruption, accelerated hypertension requiring hospitalization, and target organ-damage including cerebrovascular and cardiovascular complications. One must be cognizant of the risk for target organ damage is increased, when a sudden change in blood pressure occurs in previously normotensive women. As is true in all dynamic clinical settings, individualization of care is often the rule. In most instances, delivery of preeclamptics is indicated after 37 weeks of gestation or when fetal lung maturity has been confirmed.

### 9.4. Superimposed Preeclampsia

Superimposed preeclampsia complicates approximately 25% of pregnancies in women with chronic hypertension [102]. Principles of management are the same as outlined earlier for preeclampsia, although these women have more likelihood of developing severe hypertension, requiring multiple antihypertensive medications.

## 10. Goals of Treatment

### 10.1. Mild-to-Moderate Hypertension in Pregnancy

The benefits of antihypertensive therapy for mild to moderately increased blood pressure in pregnancy (≤160/109 mm Hg), either chronic or de novo, have not been shown in clinical trials. A Cochrane meta-analysis concluded that there are insufficient data to determine the benefits and risks of antihypertensive therapy for mild-to-moderate hypertension (defined as blood pressure 140–160 mm Hg systolic or diastolic blood pressure 90–109 mm Hg) [101]. Of note, with antihypertensive treatment there seems to be less risk of developing severe hypertension (relative risk, 0.50; with a number needed to treat of 10) but no difference in outcomes of preeclampsia, neonatal death, preterm birth, and small for gestational age babies with treatment [101]. 

International guidelines for the treatment of hypertension in pregnancy vary with respect to thresholds for starting treatment and targeted BP goals. Therapy is recommended in the United states for a BP of 160/105 mm Hg or greater [1] with no set treatment target; in Canada for women with no comorbid conditions therapy is recommended for blood pressure of 140–150/90 or greater, targeting diastolic pressure to 80–90 mm Hg and in those with comorbid conditions targeting 130–139/80–89 mm Hg [105, 106].

### 10.2. Severe Hypertension

There is consensus that treatment is indicated for severe hypertension in pregnancy, defined as 160/110 mm Hg or greater, to prevent intracerebral hemorrhage and maternal death [1, 107]. Those with hypertensive encephalopathy, hemorrhage, or eclampsia require treatment with parenteral agents to lower mean arterial pressure (two-thirds diastolic + one-third systolic blood pressure) by 25% over minutes to hours, and then to further lower blood pressure to 160/100 mm Hg over subsequent hours [1].

### 10.3. Severe Preeclampsia

Patient with severe preeclampsia are managed differently as chances for maternal and fetal complications are much higher. The criteria for diagnosis of severe preeclampsia are outlined below:

- sustained systolic blood pressure ≥160 mm of Hg;
- sustained diastolic blood pressure of ≥110 mm of Hg;
- pulmonary edema;
- oliguria <500 mL/24 hours;
- persistent headaches or scotoma;
- thrombocytopenia <100,000/mm^3^;
- persistent right upper quadrant pain or epigastric pain;
- intrauterine growth restriction <10th percentile.

The ultimate treatment in severe preeclampsia is prompt delivery. The timing of delivery is based on both maternal and fetal indications. If gestational age is less than 34 weeks, expectant management, when possible should be attempted with the aim of improving neonatal outcome without compromising the safety of the mother. This requires close inpatient monitoring of both mother and the fetus. If possible, delivery should be 48 hours after glucocorticoid administration to maximize fetal lung maturity and improve neonatal outcome. Prior to delivery, the focus is to improve placental perfusion by enhancing cardiac output and peripheral vasodilatation. Patient should be placed in lateral or supine position, which will optimize venous return and cardiac output. These patients are often intravascularly volume-depleted and require some degree of perfusion. Magnesium sulfate seizure prophylaxis is typically initiated in severe preeclamptics. The recommended dose for magnesium sulfate is 4–6 gm iv over 20 minutes followed by continuous infusion at 2 g/hr, which will maintain most patient at therapeutic range of >4 meq/mL. Blood pressure management is same as outlined above for severe hypertension with target BP of 150/100. Rapid vasodilatation with consequent hypotension should be obviated by adequate intravascular resuscitation. An absolute fetal or maternal indication for delivery requires immediate intervention. Delivery can be induced with oxytocin and prostaglandin. If induction fails, cesarean delivery is indicated.

## 11. Choice of Antihypertensive Drugs

All antihypertensive drugs cross the placenta but to variable degrees and most are agents categorized as “Category C.” There are no data from large well-designed randomized trials strong enough to mandate the use of one drug over another. Different drugs will be discussed separately based on their pharmacological actions and summarized in Table 4 [108].

### 11.1. Sympathetic Nervous System Inhibitors 

#### 11.1.1. Centrally Acting Agents

Methyldopa is one of the most widely used drugs for the treatment of hypertension in pregnancy. It is a prodrug metabolized to alpha methylnorepinephrine, which then replaces norepinephrine in the neurosecretory vesicles of adrenergic nerve terminals. BP control is gradual, over six to eight hours, because of the indirect mechanism of action. It is not thought to be teratogenic based on limited data and forty-year clinical experience. Treatment with methyldopa has been reported to prevent subsequent progression to severe hypertension in pregnancy [109] and does not seem to have adverse effects on uteroplacental or fetal hemodynamics [110].

Adverse effects are based on central alpha-2 blocking effect or decreased peripheral sympathetic tone. This drug can cause decreased mental alertness and impaired sleep, leading to sense of fatigue in some or depression in others. Still other observed side effects are decreased salivation, leading to xerostomia (chronic dry mouth), elevated liver enzymes in 5%; hepatitis and hepatic necrosis have also been reported. Some patients will develop a positive antinuclear antigen or antiglobulin (Coombs') test with chronic use, which may occasionally cause clinical hemolytic anemia.

Clonidine, a selective alpha-2 agonist, acts similarly and is comparable to methyldopa with respect to safety and efficacy [111], but of some concern is a small controlled follow-up study of twenty-two neonates that reported an excess of sleep disturbance in clonidine-exposed infants [112]. In pregnancy, it is mainly used as a third-line agent for multidrug control of refractory hypertension.

#### 11.1.2. Peripherally Acting Agents

B-blockers have been extensively used in pregnancy. Concern still remains about their safety in pregnancy based on data derived from a few small studies, which suggests an association with low birth weight infants. Atenolol, for example, in one such study started at twelve to twenty-four weeks' gestation, resulted in clinically significant fetal growth restriction and decreased placental weight compared with placebo [113, 114]. None of the b-blockers have been associated with teratogenicity. Oral agents have been associated with nonclinically significant neonatal bradycardia. Parenteral therapy has been found to increase the risk of neonatal bradycardia in one of six newborns. Results from one-year follow-up study, which showed normal development of infants exposed to atenolol in utero, are reassuring [115]. In a recent Cochrane analysis, b-blockers were found to be more effective in lowering BP than methyldopa in ten trials. 

Labetolol, a nonselective beta- and alpha-blocker has gained wide acceptance in pregnancy. Oral administration is considered safe and effective as methyldopa [116, 117], although neonatal hypoglycemia is reported at high doses. Based on one placebo trial, it has been associated with growth restriction when used in management of preeclampsia remote from term. Parenterally it is used to treat severe hypertension, and because of a lower incidence of maternal hypotension and other side effects, many find this drug preferable to hydralazine. Reported adverse events are fatigue, lethargy, exercise intolerance, peripheral vasoconstriction, sleep disturbance, and bronchoconstriction. It has moved up to the category of a first-line agent in the opinion of many clinicians.

Peripherally acting alpha-adrenergic antagonists are second-line antihypertensive drugs in nonpregnant adults. These are indicated during pregnancy in the management of hypertension in patients suspected to have pheochromocytoma. Both prazosin and phenoxybenzamine have been used, with b-blockers used as adjunctive agents after alpha-blockade is accomplished [118, 119]. There is only limited experience with these agents in pregnancy; therefore, their routine use cannot be advocated.

### 11.2. Calcium Channel Blockers

These drugs have been used to manage chronic hypertension, mild pre-eclampsia presenting late in gestation and urgent hypertension associated with preeclampsia. Both nifedipine a nondihydropyridine calcium channel blocker and verapamil are not associated with teratogenic risks to fetus exposed in first trimester [120]. Maternal adverse effects with nifedipine include tachycardia, palpitations, peripheral edema, headaches, and facial flushing. Nifedipine does not seem to cause a detectable decrease in uterine blood flow [121, 122]. Short-acting dihydropyridine calcium antagonists, particularly when administered sublingually, are now not recommended for the treatment of hypertension in nonpregnant patients because of reports of myocardial infarction and death in hypertensive patients with coronary artery disease. In pregnant patients, these formulations are associated with maternal hypotension and fetal distress [123, 124] and are generally not recommended. In contrast long-acting oral nifedipine in pregnant patients with severe hypertension in pregnancy has been shown to be safe and effective [125]. Dihydropyridine compounds also have a tocolytic effect and can delay the onset or slow the progression of labor. Nondihydropyridine agents such as verapamil and diltiazem may have added value in women with proteinuria because of their antiproteinuric action.

A concern with the use of calcium antagonists for BP control in preeclampsia has been the concomitant use of magnesium sulfate to prevent seizures. Drug interactions between nifedipine and magnesium sulfate were reported to cause neuromuscular blockade, myocardial depression, or, in some cases, circulatory collapse. Despite this concern, recent evaluation showed that these medications are commonly used together without increased risk.

### 11.3. Diuretics

Diuretics are first-line agents to be used in management of essential hypertension prior to conception and, based on their apparent safety, they may be continued during pregnancy alone or in combination with other agents especially in women more likely to have salt-responsive hypertension [1]. Concerns regarding volume contraction leading to limited fetal growth have not been supported in studies [126]. Mild volume contraction, however, may lead to hyperuricemia and in doing so invalidate serum uric acid levels as a laboratory marker that may assist in the diagnosis of superimposed preeclampsia. The adverse effects are mainly due to fluid, electrolytes, and solute disturbances.

In women already taking, hydrochlorothiazide may be continued during pregnancy; use of low-doses (12.5 to 25 mg daily) may minimize untoward metabolic effects, such as impaired glucose tolerance and hypokalemia [127]. The potassium sparing diuretics triamterene and amiloride do not appear to be teratogenic based on a small numbers of case reports [127]. On the other hand, spironolactone is not recommended because of its antiandrogenic effects during fetal development, noted in animal models, although this concern was not borne out in an isolated clinical case, where this agent was employed [128].

### 11.4. Direct Vasodilators

Hydralazine: selectively relaxes arteriolar smooth muscle by an as yet unknown mechanism. The most important indication is severe hypertension or a third-line agent in control of refractory hypertension. It can be used orally, intravenously, or intramuscularly. Adverse effects are due to excessive vasodilation or sympathetic activation (headache, nausea, flushing, or palpitations). Chronic use can lead to (in rare cases) a pyridoxine-responsive polyneuropathy or to immunologic reactions, including a drug-induced lupus syndrome. Hydralazine has been used in all trimesters of pregnancy, and data have not shown an association with teratogenicity, although neonatal thrombocytopenia and lupus have been reported [129]. For acute severe hypertension later in pregnancy, intravenous hydralazine has been associated with more maternal and perinatal adverse effects than intravenous labetalol or oral nifedipine. Maternal hypotension, cesarean sections, placental abruptions, Apgar scores less than 7, and oliguria occur more frequently following hydralazine. A recent meta-analysis of the use of intravenous hydralazine in severe hypertension in pregnancy concluded that parenteral labetalol or oral nifedipine were preferable first-line agents, with hydralazine as a suitable second-line agent [130].

#### 11.4.1. Isosorbide Dinitrates

this agent has been investigated in a small study of gestational hypertensive and preeclamptic pregnant patients. It was found that cerebral perfusion pressure is unaltered by isosorbide dinitrate, despite significant changes in maternal BP, thus decreasing the risk for ischemia and infarction, when BP is lowered [131].

#### 11.4.2. Sodium Nitroprusside

This direct nitric oxide donor, which relaxes both arteriolar and venular vascular smooth muscles. It is used only as continuous infusion and is easily titrated because of its near immediate action of onset and only three-minute duration of action. Nitroprusside metabolism releases cyanide, which can reach toxic levels when infused rapidly. Cyanide metabolizes to thiocyanate which can lead to toxicity in 24–48 hours. Adverse effects include excessive vasodilation and cardioneurogenic (i.e., paradoxical bradycardia) syncope in volume-depleted preeclamptic women [132].

 N.B.: The risk of fetal cyanide intoxication remains unknown. Given the long experience with hydralazine and alternative use of parenteral labetalol or oral calcium channel blockers, this drug is considered as a last resort.

### 11.5. Serotonin Receptor Blockers

Serotonin-induced vasodilation is mediated by S1 receptors and subsequent release of prostacyclin and NO. Ketanserin is a selective S2 receptor-blocking agent that has been used in the nonpregnant population. Based on the data available from Australia and South Africa, it may be safe to use in pregnancy and useful in treatment of chronic hypertension in pregnancy, preeclampsia, and HELLP syndrome [133, 134]. FDA has not approved Ketanserin for use in United States.

### 11.6. Angiotensin-Converting Enzyme Inhibitors (ACE-I) and Angiotensin Receptor Antagonists (ARB)

ACE-I and ARB are contraindicated in 2nd and 3rd trimesters because of severe toxicity secondary to reduced renal perfusion of the fetal kidneys. Their use has been associated with renal dysgenesis, oligohydramnios as a result of fetal oliguria, calvarial and pulmonary hypoplasia, intrauterine growth restriction, and neonatal anuric renal failure, leading to death of the fetus [135, 136]. ARBs have also been associated with fetal demise and same concerns are applicable to the use of direct renin inhibitors. First trimester exposure to these agents has been associated with greater incidence of cardiovascular and central nervous system malformations. Whether these effects are secondary to hemodynamic effects or specific requirement of angiotensin II as a fetal growth factor is unknown. Patients should, therefore, be counseled to stop these medications while attempting to conceive. The risk of birth defects increased from 3 to 7% while on these medications at the time of conception [137].

## 12. Postpartum Hypertension

Some women experience a rise in their BP in the postpartum period, which typically reaches a maximum on the fifth postpartum day. This has been attributed to volume expansion and fluid mobilization in postpartum period. The time period, beyond which patients with gestational hypertension and preeclampsia are labeled chronic hypertensive, is not well defined. The threshold for treatment in these patients has not been uniformly established, but medication is generally recommended to be started when systolic BP exceeds 150 mm Hg or diastolic BP is greater than 100 mm of Hg [138]. The choice of antihypertensive agents is, of course, influenced by whether or not the patient is breast-fed. In selected patients with severe preeclampsia, especially those with hypertension accompanied by peripheral and pulmonary edema, a short course of loop diuretics may be beneficial.

NSAIDS may contribute to postpartum hypertension as per few case reports [139], and thus their use should be avoided in patients who are already hypertensive.

## 13. Breast Feeding

Neonatal exposure to methyldopa via nursing is likely low and is generally considered safe. Atenolol and metoprolol are concentrated in breast milk, possibly to levels that could affect the infant; by contrast, exposure to labetolol and propanolol appears low [140]. Although milk concentrations of diuretics are low and considered safe, these agents, by inducing volume contraction in mother, can decrease milk production [141]. There are reports of calcium channel blockers transfer into breast milk [142], but the relative infant dose of nifedipine, verapamil, and diltiazem is low, and all are safe during breast-feeding. Sufficient data exist for the safety of three ACE inhibitors: captopril, enalapril, and quinapril; these drugs are deemed to be compatible with breast-feeding by the American Academy of Pediatrics [143]. There are currently insufficient data on angiotensin II receptor blockers and at this time the recommendation is not to use these drugs during breast-feeding.

## Figures and Tables

**Table 1:** Classification of hypertension in pregnancy.

Chronic hypertension	(i) increased BP before week 20 (or known to exist prior to pregnancy) (ii) hypertension persistent for more than 12 weeks after pregnancy

Preeclampsia-eclampsia	(i) de novo appearance of hypertension after mid-pregnancy (ii) proteinuria at least 300 mg/24 hr

Preeclampsia superimposed upon existing hypertension	(i) new onset proteinuria

Gestational hypertension	(i) transient hypertension appearing after mid-pregnancy (ii) confirmed by return to normal BP postpartum (iii) no proteinuria

**Table 2:** Laboratory tests of renal function during pregnancy.

	Nonpregnant	Pregnant
BUN	13 ± 3	8.17 ± 1.5
Creatinine	0.67 ± 0.14	0.46 ± 0.13
Creatinine clearance	80–120	125
Serum uric acid	Greater than 4	Less than 4
Urinalysis	Normal	Normal

**Table 3:** Observations supporting uteroplacental ischemia as a key factor in preeclampsia.

(i) predominantly occurs in primagravidas with immature uterine vasculature
(ii) consistent abnormalities of the placentae and uteroplacental vascular interface
(iii) increased risk with more fetuses and placentas (twins)
(iv) disease occurs late in gestation
(v) labor aggravates
(vi) high incidence with large, rapidly growing hydatidiform moles
(vii) increase incidence in patients with underlying vascular disease (diabetes, hypertension and lupus (SLE))
(viii) findings in animals subjected to uteroplacental ischemia mimic those of preeclampsia

**Table 4:** Antihypertensives in pregnancy.

Drugs	Method of action (MOA)	Side effects	Fetal concerns	Indication	Dosage
*(I) Sympathetic nervous system inhibitors *					
(A) Central acting					
(1) Methyldopa agent of choice	Alpha2 agonist. Onset is gradual (6–8 hrs)	Decreased mental alertness, impaired sleep, sense of fatigue and depression, xerostomia	Considered safe (Category B)	Preferred drug for non emergent BP control	0.5–3 gm PO in 2 divided doses
(2) Clonidine	Alpha-2 agonist	As above	Limited data (Category C). Considered safe as same MOA as methyldopa	Nonemergent BP control	to 0.3 mg q8–12 hrs
(B) Peripheral acting					
(1) Labetalol	Beta and alpha blocker	Fatigue, lethargy, exercise intolerance, sleep disturbances, and asthma	Concern for LBW infants and decrease uteroplacental blood flow though long-term data suggesting safety. Risk of neonatal hypoglycemia at higher doses (Category C)	Nonemergent and emergent Bp control	20–1200 mg in 2-3 divided doses. 10–20 mg IV then 20–80 mg IV every 20–30 minutes, maximum of 300 mg: for infusion 1-2 mg/min

*(II) Calcium channel blockers*					
(A) Nifedipine	Calcium channel blocker Dihydropyridine	Tachycardia, palpitations, peripheral edema, headache, and facial flushing	Does not cause decrease maternal blood flow. Concern regarding concomitant uses with magnesium though not proven in recent evaluations (Category C)	Non-emergent and emergent BP control	30–120 mg extended release preparations

*(III) Direct vasodilators *					
(A) Hydralazine	Selectively relaxes arteriolar smooth muscle by an unknown mechanism	Acutely: Headache, nausea, flushing, palpitations. Chronic use: Pyridoxine-responsive polyneuropathy or immunologic reaction like drug induced lupus	Effect on uteroplacental blood flow is uncertain. Associated with more maternal and perinatal adverse events than other agents when used acutely. Neonatal thrombocytopenia and Lupus have been reported. Not proven to be teratogenic	Useful in combination with sympatholytic agents. Used for emergent and nonemergent BP control	50–300 PO mg in 2–4 divided doses. 5 mg IV, then 5–10 mg every 20–40 min: once BP controlled repeat every 3 hours. For infusion: 0.5 to 10 mg/hr
(B) Sodium nitroprusside					
Relatively contraindicated. Considered as a last resort	Direct NO inhibitor Non-selectively relaxes arteriolar and venular vascular smooth muscles	Excessive vasodilation and cardioneurogenic syncope in volume depleted patients. Cyanide toxicity if used greater than 4 hours. Labor arrest hyperglycemia	Risk of fetal cyanide intoxication remains unknown	Only for emergent Bp control as last resort	30–50 mg IV every 5–15 minutes Infusion at 0.25–5.0 mcg/kg/min
*(IV) Diuretics *					
(A) Hydrochlorothiazide	Thiazide diuretics. Blocks Na channels in distal-convoluted tubules	Volume contraction and electrolyte disturbances. Hyperuricemia	Volume contraction may limit fetal growth, though not proven in studies	Nonemergent BP control	12.5–25 mg/day

*(V) ACE-I and ARB *					
Contraindicated in pregnancy. Should be discontinued prior to conception	ACE-I: inhibits angiotensin-converting enzyme interfering with conversion of angiotensin I to angiotensin II ARB: antagonizes ATI receptor	Angioedema, hypotension, hyperkalemia, renal failure. Cough (only in ACE-I)	ACE-I: renal dysgenesis, oligohydramnios, calvarial and pulmonary hypoplasia, IUGR. Neonatal anuric renal failure, fetal death Arbs: same concerns	Not indicated	N/A
